# User Experience of a Computer-Based Decision Aid for Prenatal Trisomy Screening: Mixed Methods Explanatory Study

**DOI:** 10.2196/35381

**Published:** 2022-09-06

**Authors:** Titilayo Tatiana Agbadje, Chantale Pilon, Pierre Bérubé, Jean-Claude Forest, François Rousseau, Samira Abbasgholizadeh Rahimi, Yves Giguère, France Légaré

**Affiliations:** 1 VITAM - Centre de recherche en santé durable Centre intégré universitaire de santé et services sociaux de la Capitale-Nationale Quebec, QC Canada; 2 Greybox Solutions Inc Montreal, QC Canada; 3 Department of Molecular Biology, Medical Biochemistry, and Pathology Faculty of Medicine Université Laval Quebec, QC Canada; 4 Department of Family Medicine Faculty of Medicine McGill University Montreal, QC Canada; 5 Lady Davis Institute for Medical Research Jewish General Hospital Montreal, QC Canada; 6 Department of Family Medicine and Emergency Medicine Faculty of Medicine Université Laval Quebec, QC Canada

**Keywords:** shared decision-making, computer-based decision aid, prenatal screening, trisomy, Down syndrome, mixed methods

## Abstract

**Background:**

Mobile health tools can support shared decision-making. We developed a computer-based decision aid (DA) to help pregnant women and their partners make informed, value-congruent decisions regarding prenatal screening for trisomy.

**Objective:**

This study aims to assess the usability and usefulness of computer-based DA among pregnant women, clinicians, and policy makers.

**Methods:**

For this mixed methods sequential explanatory study, we planned to recruit a convenience sample of 45 pregnant women, 45 clinicians from 3 clinical sites, and 15 policy makers. Eligible women were aged >18 years and >16 weeks pregnant or had recently given birth. Eligible clinicians and policy makers were involved in prenatal care. We asked the participants to navigate a computer-based DA. We asked the women about the usefulness of the DA and their self-confidence in decision-making. We asked all participants about usability, quality, acceptability, satisfaction with the content of the DA, and collected sociodemographic data. We explored participants’ reactions to the computer-based DA and solicited suggestions. Our interview guide was based on the Mobile App Rating Scale. We performed descriptive analyses of the quantitative data and thematic deductive and inductive analyses of the qualitative data for each participant category.

**Results:**

A total of 45 pregnant women, 14 clinicians, and 8 policy makers participated. Most pregnant women were aged between 25 and 34 years (34/45, 75%) and White (42/45, 94%). Most clinicians were aged between 35 and 44 years (5/14, 36%) and women (11/14, 79%), and all were White (14/14, 100%); the largest proportion of policy makers was aged between 45 and 54 years (4/8, 50%), women (5/8, 62%), and White (8/8, 100%). The mean usefulness score for preparing for decision-making for women was 80/100 (SD 13), and the mean self-efficacy score was 88/100 (SD 11). The mean usability score was 84/100 (SD 14) for pregnant women, 77/100 (SD 14) for clinicians, and 79/100 (SD 23) for policy makers. The mean global score for quality was 80/100 (SD 9) for pregnant women, 72/100 (SD 12) for clinicians, and 80/100 (SD 9) for policy makers. Regarding acceptability, participants found the amount of information just right (52/66, 79%), balanced (58/66, 88%), useful (38/66, 58%), and sufficient (50/66, 76%). The mean satisfaction score with the content was 84/100 (SD 13) for pregnant women, 73/100 (SD 16) for clinicians, and 73/100 (SD 20) for policy makers. Participants thought the DA could be more engaging (eg, more customizable) and suggested strategies for implementation, such as incorporating it into clinical guidelines.

**Conclusions:**

Pregnant women, clinicians, and policy makers found the DA usable and useful. The next steps are to incorporate user suggestions for improving engagement and implementing the computer-based DA in clinical practice.

## Introduction

Pregnant women and their partners must decide whether to undergo prenatal screening to assess the risk of certain genetic conditions (eg, the presence of Down syndrome) in the fetus [1]. However, they may be unaware of the implications of the various options, unclear about which implications matter most, or unaware that they can choose not to do the test at all [1]. The decision regarding screening is complex as it may lead to other difficult decisions (eg, pregnancy termination) [2]. Thus, pregnant women and their partners have numerous decisional needs regarding prenatal screening that are rarely addressed by health care systems [1]. As a result, many experience decisional conflicts (discomfort with the decision made), which may later translate into decision regret [3].

Shared decision-making (SDM) is both a patient-centered philosophy of care and a process whereby clinicians engage patients as partners to make choices about care based on clinical evidence and patients’ values and preferences [4]. This fosters both informed consent and patient empowerment [1,5-7]. In deciding about prenatal screening, SDM seems a promising approach to supporting women and their partners, as it is a preference-sensitive decision (ie, one for which there is no “best choice”). To support women in these decisions, physicians must solicit patients’ values and preferences and communicate probabilistic evidence in an understandable manner. Women and clinicians are both usually willing to engage in SDM but require effective decision support tools. This is especially true for women with less education, who exhibit lower decision self-efficacy (self-confidence about decision-making) [8]. Therefore, there are increasing calls for improving strategies for communicating risks and benefits, and for deliberation tools such as decision aids (DAs) [9].

DAs provide a detailed, specific, and personalized focus on options and outcomes to prepare people for decision-making before or between consultations with their physicians [7]. They can be in the form of brochures, booklets, webpages, or apps that provide users with information and help clarify their values and preferences regarding options [7,10-12]. They have been shown to be effective in increasing knowledge, patient-clinician communication, and the use of options that are beneficial to most while reducing the overuse of options that are not beneficial [7]. In pregnancy care, the use of DAs has shown positive effects on informed decision-making [13] and is associated with more value-congruent choices [14].

Computer-based DAs, such as in the form of an app, have the advantage of being accessible to people on their digital devices, can be customized to fit the needs of users, and can automatically integrate the latest medical evidence. Mobile health (mHealth) apps such as computer-based DAs have been shown to have a favorable impact on SDM and patient satisfaction with patient-clinician interactions [15].

We recently developed a computer-based DA for prenatal screening in partnership with a commercial mHealth firm [16]. In preparation for a large-scale rollout, we sought to assess its usability and usefulness among pregnant women, clinicians, and policy makers.

## Methods

### Study Design and Settings

This mixed methods sequential explanatory study pilot-tested the new computer-based DA. For reporting, we used the Mixed Methods Article Reporting Standards (MMARS) [17] and the Standards for Universal reporting of patient Decision Aid Evaluation studies (SUNDAE) checklists (Multimedia Appendix 1) [18].

### Participants and Recruitment

The participants were pregnant women, clinicians, and policy makers. Pregnant women were recruited at 3 clinical sites in Quebec City: (1) the *Maison des naissances de la Capitale-Nationale* (a birthing center), (2) the Obstetrics and Gynecology Department at the *Centre hospitalier universitaire de Québec*, and (3) the Family Medicine Unit at St-François d’Assise Hospital. Approval for recruitment was obtained from each clinical site manager. A research assistant and students recruited pregnant women in the waiting rooms of the participating sites. Clinicians involved in prenatal care were recruited from the same birthing center and 2 other clinical sites in Lévis, Quebec: the Obstetrics and Gynecology Department of the Hôtel-Dieu de Lévis Hospital and the *Maison des naissances Mimosa*. We identified policy makers from the organigrams of organizations and institutions interested in prenatal screening (eg, Quebec’s Ministry of Health, its public health authority, and a rehabilitation centre, the *Institut de réadaptation en déficience physique de Québec*) and contacted them by email. Clinicians and policy makers were also recruited from professional and social networks using the snowball sampling method.

### Eligibility Criteria

As we did not want to interfere with the outcome of their decisions regarding prenatal screening, we recruited women who had already made the decision to answer questions about the DA. Women in Quebec make the decisions at 16 weeks of pregnancy. Therefore, eligible women had to (1) be >16 weeks pregnant or have given birth in the previous year, (2) have made a decision about prenatal screening for trisomy, and (3) be aged at least 18 years. A cutoff of 1 year was chosen to minimize the forgetting bias effect. We excluded women who had participated in previous studies on prenatal screening conducted by our team [19-21]. We also excluded women who presented with a high-risk pregnancy (eg, pre-eclampsia, gestational diabetes, or multiple pregnancies) because of ethical considerations. High-risk pregnancies can be emotionally distressing and accompanied by physical disabilities. Eligible clinicians were involved in prenatal screening, and eligible policy makers had decisional responsibilities in the health and social services sector. All participants had to be able to speak and write in French or English and be able to give informed consent.

### Computer-Based DA

On the basis of a validated paper-based DA [22], the computer-based DA was developed and tested by the project leader and 2 professionals from Greybox Solutions Inc. It is available on their platform [16]. The computer-based DA menu has 5 tabs: home, trisomy, tests, test comparison, and questionnaire. The home page outlines the options available and provides advice on how to make informed decisions. The trisomy page presents information on trisomy 21, 18, and 13, as well as the main risk factor (maternal age) and the estimated risk of trisomy by maternal age in a population of 10,000 pregnant women. The tests page presents details on screening and diagnostic tests for trisomy available in the province of Quebec. The comparison page compares the overall performance of different tests or combinations of tests (eg, sensitivity and specificity). The questionnaire page includes a values and preferences clarification exercise to help users consider what matters most about the benefits and risks of the options. It has 5 subtabs. The first presents the benefits and risks of performing or not performing a test (any test). It also has empty boxes where users can enter the benefits and risks not included in our list. Users rate the importance they attach to each benefit and risk on a scale of 0=not important to 10=very important. The second subtab summarizes the user’s benefit and risk assessment for performing a test or not performing a test (any test) to help them decide whether to take the test. If they decide to take the test, the third subtab provides information on the available tests for comparison purposes based on the week of pregnancy at which the test can be done, waiting time for results, detection rate, accuracy, potential cost, and other factors (to be completed by the user), whose importance is rated by the users on a scale from 0 to 10. The fourth subtab provides a summary of the importance that users had assigned to each factor to help them select the best test. Once they have made their selection, the fifth subtab presents the *SURE* test for evaluating the person’s certainty about the decision made (Sure of myself, understand information, risk-benefit ratio, and encouragement) [3]. On this page, users may enter their email address and receive a summary of their answers to be discussed later with their partners and accompanying clinicians. The computer-based DA is available in French and English.

### Data Collection

#### Overview

Meetings with pregnant women lasted for approximately 45 minutes. First, the research assistant invited women to participate and, if they agreed, presented the study details and collected signed consent forms. Subsequently, women were each given a tablet (iPad Wi-Fi, 6th Generation, model 1893) with a link to the computer-based DA, which they could navigate at their leisure. There were no specific instructions, and research professionals were available to answer questions. Women then self-completed a questionnaire on sociodemographics (including the partner’s), perceived usefulness, self-efficacy, usability, quality, acceptability, and satisfaction with the content in their own time. A total of 3 main objectives directed the choice of variables. First, based on the social learning theory [23], we used variables that would inform us about whether the DA would give women the self-confidence (self-efficacy) to make a health decision and whether it prepared them adequately to meet with a health provider to make an informed, value-congruent decision (perceived usefulness), in line with the goals of SDM. Second, using scales developed specifically for digital tools, we examined women’s perceptions of whether the DA was efficient, easy, and enjoyable to use (usability and quality). Third, we sought perceptions more specifically of the DA content; that is, variables such as comprehensibility, presentation (eg, balance), and length (acceptability and satisfaction with content). Finally, participants were interviewed, and their experiences with computer-based DA were audio-recorded. The questionnaire and interview guide were reviewed in a team meeting, and, in keeping with the comments received, questions were reformulated for better comprehension. The participating women received compensation for CAD $40 (US $30.7). Data collection for clinicians and policy makers was different: recruits were contacted by email and asked to sign and return a consent form to begin the study. After receiving consent, we emailed them the link to the computer-based DA and the questionnaire. They tried the computer-based DA, filled out the questionnaire at their own pace, and emailed it back. We then scheduled a 15-minute audio-recorded interview at a time that suited them. Clinicians and policy makers did not receive any compensation for their participation. Meetings took place either at our research center or at the participants’ place of choice (home or workplace).

#### Outcomes and Measures

We assessed perceived usefulness and decision self-efficacy among women using the Preparation for Decision-Making scale [24] and the Decision Self-Efficacy scale [25], respectively. We assessed perceptions among women, clinicians, and policy makers regarding the usability, quality, and acceptability of the computer-based DA and their satisfaction with its content using the System Usability Scale [26,27], the user version of the Mobile App Rating Scale (uMARS) [28], the acceptability questionnaire by O’Connor and Cranney [29], and a self-developed satisfaction with the content questionnaire (Table 1). The perspectives of clinicians and policy makers on these outcomes are important as SDM is a 2-way process, with clinicians sharing evidence and patients reflecting their life experiences, preferences, and values. Clinicians and policy makers are also likely to be involved in integrating DAs into clinical pathways and protocols. The satisfaction questionnaire was developed by our team and, therefore, was not validated. We asked participants to rate (*disagree very much* to *agree very much*) whether they were satisfied with the content of the computer-based DA on a 5-point scale. Specifically, we asked participants whether they were satisfied with the information regarding the prevalence and description of trisomy 21, screening tests, risks associated with each screening test, advantages and disadvantages of each screening option, and preferences and decisional comfort.

**Table 1:** Variables and measurement tools.

Variable	Measurement tool	Authors	Purpose	Number of items, scale	Example of question	Psychometric properties in the literature	Psychometric properties in the study sample
Perceived usefulness	Preparation for Decision-Making scale	Graham and O’Connor [24]	Evaluates how useful the computer-based DA^a^ is in preparing participants to communicate about the decision with their practitioner in a consultation	10 items, 5-point Likert scale (1=strongly disagree to 5=strongly agree)	“Did this educational material help you think about which pros and cons are most important?”	Cronbach α ranging from .92 to .96	Cronbach α of .85 for pregnant women
Self-efficacy	Decision Self-Efficacy scale	O’Connor [25]	Measures self-confidence or belief in one’s abilities of decision-making, including shared decision-making	11 items, 5-point Likert scale (1=not at all confident to 5=very confident)	“I feel confident that I can get the facts about the choices available to me”	Cronbach α coefficient of .92	Cronbach α of .88 for pregnant women
Usability of the computer-based DA	System Usability Scale	Brooke [30]	Used to improve prototype mobile technologies by measuring preliminary needs of users, user experience, and usability, including the efficacy and satisfaction with which users accomplish specific tasks	10 items, 5-point Likert scale (1=strongly disagree to 5=strongly agree)	“I thought there was too much inconsistency in this system.”	Cronbach α coefficient of .91	Cronbach α of .88 for pregnant women and .87 for clinicians
Quality of the computer-based DA	User version of the Mobile App Rating Scale	Stoyanov et al [28]	Measures the quality of an app through its 5 criteria categories: entertaining (whether the app is fun or entertaining to use), interest (whether it is interesting to use), customization (whether it allows the customization of settings and preferences), interactivity (whether it allows user input, provides feedback, and contains prompts), target group (whether its content is appropriate for the target audience)	20 items, 5-point Likert scale (1=inadequate, 2=poor, 3=acceptable, 4=good, and 5=excellent)	“Entertainment: Is the app fun or entertaining to use? Does it have components that make it more fun than other similar apps? 1) Dull, not fun or entertaining at all; 2) Mostly boring; 3) OK, fun enough to entertain user for a brief time (<5 minutes); 4) Moderately fun and entertaining, would entertain user for some time (5-10 minutes total); 5) Highly entertaining and fun, would stimulate repeat use.”	Cronbach α=.90	Cronbach α of .61 for pregnant women and .75 for clinicians
Acceptability of the computer-based DA	Acceptability questionnaire	O’Connor and Cranney [29]	Evaluate the comprehensibility of components, length, amount of information, sufficiency of information, balance in option presentation, and overall suitability for decision-making through structured and semistructured questions	10 items, variable (2-4 choices of answers for the structured questions)	“The amount of information was: 1) too much information; 2) too little information; 3) just right.”	N/A^b^	N/A
Satisfaction with the content of the computer-based DA	Satisfaction questionnaire developed based on the literature	Self-developed	Each item related to a specific page of the computer-based DA	6 items, 5-point Likert scale (1=strongly disagree to 5=strongly agree)	“I am satisfied with the information on the various screening tests for Trisomy 21.”	Not validated	Cronbach α of .77 for pregnant women and .83 for clinicians

^a^DA: decision aid.

^b^N/A: not applicable.

#### Interview Guide

We qualitatively explored the participants’ reactions to the computer-based DA and solicited their suggestions. We developed a dynamic interview guide based on the uMARS scale and its subscales (engagement, functionality, aesthetics, information, and global evaluation) [28]. After the participants completed the questionnaire, the research assistant selected items receiving a poorer evaluation (ie, a rating of 1=inadequate, 2=poor, or 3=acceptable) and asked for explanations and suggestions for improvement. When all items received a good rating, the research assistant asked general questions, as well as suggestions for improvement. Clinicians and policy makers were also asked for ideas about implementing the DA. The interviews were conducted by a research professional assisted by a trainee or student who took notes.

### Sample Size

We recruited a purposive sample of pregnant women, clinicians, and policy makers. We recruited identical sample sizes for the quantitative and qualitative phases [31]. Using data from a study involving 60 in-depth interviews, Guest and al [32] found that data saturation occurred within the first 12 interviews. Thus, we planned to recruit up to 15 pregnant women per clinical site (a total of 45 women), 15 clinicians per clinical site (a total of 45 clinicians), and 15 policy makers until data saturation was achieved. This translated into a total of 105 participants.

### Data Analysis

We used descriptive statistics (means, SDs, percentages, and 95% CIs) for the sociodemographic characteristics and quantitative variables. Quantitative analyses were performed using SAS (version 9.4; SAS Institute). We proceeded to imputation using means to treat the missing data.

The interviews were audio recorded and transcribed verbatim. The transcripts were checked by a second individual. We performed deductive and inductive thematic analyses of the transcripts using qualitative data analysis software (NVivo, version 12, QSR International). We used the Braun and Clarke [33] step-by-step guide to conducting thematic analyses. Qualitative analysts independently read the transcripts to familiarize themselves with the data and attached initial codes according to the most basic elements of the raw data. Coding was performed by TTA, CP, and 2 trainees. They then met to cross-check their coding and analyzed the categories and links between them. Discrepancies were discussed until a consensus was reached.

### Ethics Approval and Consent to Participate

This project, entitled “TechnOlogy assisted PrenaTal screEning deCisions,” was approved by the ethics committee of the Centre hospitalier universitaire de Québec-Université Laval (MP-20-2019-4451) and the Centre intégré de santé et de services sociaux de Chaudière-Appalaches (MEO-20-2019-632). The project was described to eligible participants, and they were informed that the data were anonymous and confidential. Those who wished to participate provided written informed consent.

## Results

### Participants’ Characteristics

**Figure 1:**
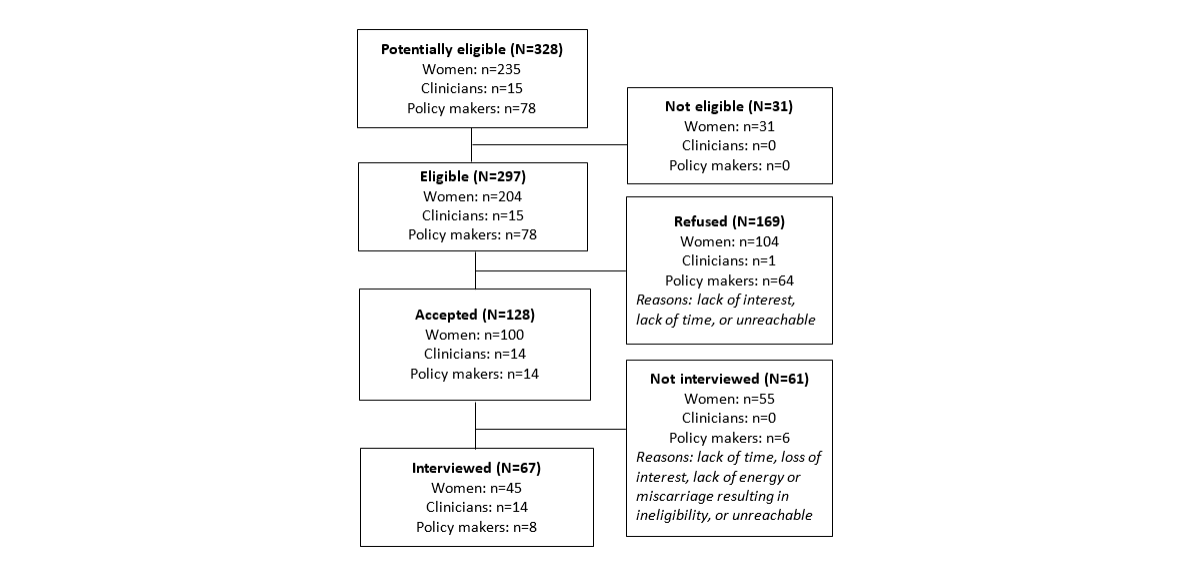
Flowchart of participants: pregnant women, clinicians, and policy makers.

From February 2019 to May 2020, a total of 328 participants were approached, of whom 297 (90.5%) were eligible, 169 (51.5%) declined to participate, and 128 (39%) agreed to participate. Of these 128 participants, 67 (52.3%) were interviewed, including 45 (35.1%) pregnant women, 14 (10.9%) clinicians, and 8 (6.2%) policy makers (Figure 1). Participants who declined to participate cited a lack of interest or time. Participants who canceled their participation mentioned a lack of time, loss of interest, lack of energy, or miscarriage resulting in ineligibility. The others were either unreachable or did not respond to calls or messages.

Most pregnant women participating in the study were aged between 25 and 34 years (34/45, 75% women, and 23/45, 51% partners), White (42/45, 94% women, and 40/45, 89% partners), and university educated (29/45, 64% women, and 25/45, 56% partners), and had a relatively high socioeconomic status (21/45, 47% had an annual family income of ≥CAD $100,000 [US $76,875]; Table 2).

Most clinicians were aged between 35 and 44 years (5/14, 36%), women (11/14, 79%), and White (14/14, 100%). Four types of clinicians participated in this study: midwives (7/14, 50%), family physicians (3/14, 21%), gynecologist-obstetricians (3/14, 21%), and neonatologists (1/14, 7%). The average number of years of experience was 13.3 (SD 11.5) years, and the average number of pregnancy follow-ups per week was 10.4 (SD 9; Table 2).

Most policy makers were aged between 45 and 54 years (4/8, 50%), women (5/8, 62%), and White (8/8, 100%). The 8 policy makers included 2 (25%) managers, 2 (25%) socioeconomic research and planning officers, 2 (25%) researchers, 1 (12%) assistant director, and 1 (12%) expert advisor. The mean number of years of experience was 17 (SD 9.5) years (Table 2).

**Table 2:** Participant characteristics (N=67).

Characteristics		Women (n=45)	Partners (n=45)	Clinicians (n=14)	Policy makers (n=8)
**Age (years), n (%)**					
	18-24	3 (7)	0 (0)	0 (0)	0 (0)
	25-34	34 (75)	23 (51)	3 (21)	2 (25)
	35-44	7 (16)	16 (36)	5 (36)	0 (0)
	45-54	1 (2)	1 (2)	1 (7)	4 (50)
	55-64	0 (0)	0 (0)	4 (29)	2 (25)
	Missing data	0 (0)	5 (11)	1 (7)	0 (0)
**Gender, n (%)**					
	Woman	N/A^a^	2 (5)	11 (79)	5 (62)
	Man	N/A	42 (93)	3 (21)	3 (38)
	Other	N/A	0 (0)	0 (0)	0 (0)
	Missing data	N/A	1 (2)	0 (0)	0 (0)
**Ethnicity, n (%)**					
	White	42 (94)	40 (89)	14 (100)	8 (100)
	African or African American	1 (2)	2 (5)	0 (0)	0 (0)
	Indigenous	1 (2)	1 (2)	0 (0)	0 (0)
	Asian	0 (0)	1 (2)	0 (0)	0 (0)
	Other	1 (2)	0 (0)	0 (0)	0 (0)
	Missing data	0 (0)	1 (2)	0 (0)	0 (0)
**Language, n (%)**					
	French	44 (98)	41 (90)	14 (100)	8 (100)
	English	0 (0)	2 (5)	0 (0)	0 (0)
	Other	1 (2)	0 (0)	0 (0)	0 (0)
	Missing data	0 (0)	2 (5)	0 (0)	0 (0)
**Residency status, n (%)**					
	Canadian	39 (87)	39 (87)	14 (100)	7 (88)
	Permanent or temporary resident	6 (13)	4 (9)	0 (0)	1 (12)
	Missing data	0 (0)	2 (4)	0 (0)	0 (0)
**Civil status, n (%)**					
	Single	10 (22)	10 (22)	—^b^	—
	Married or in a common law relationship	34 (76)	32 (71)	—	—
	Separated	1 (2)	1 (2)	—	—
	Missing data	0 (0)	2 (5)	—	—
**Education, n (%)**					
	Elementary school	0 (0)	1 (2)	—	—
	High school or professional diploma	6 (14)	13 (29)	—	—
	College diploma	10 (22)	5 (11)	—	—
	University, bachelor’s degree, or equivalent	15 (33)	15 (33)	—	—
	University, master’s degree, or equivalent	12 (27)	8 (18)	—	—
	University or PhD	2 (4)	2 (5)	—	—
	Other	0 (0)	1 (2)	—	—
**Annual family income (CAD $ [US $]), n (%)**					
	<29,999 (<23,061)	1 (2)	1 (2)	—	—
	30,000-59,999 (23,062-46,123)	4 (9)	4 (9)	—	—
	60,000-99,999 (46,124-76,873)	19 (42)	19 (42)	—	—
	≥100,000 (≥76,874)	21 (47)	21 (47)	—	—
Years of experience, mean (SD; range)		N/A	N/A	13.3 (11.5; 1.5-36)	17 (9.5; 1.5-30)
Number of pregnancy follow-ups per week, mean (SD; range)		N/A	N/A	10.4 (9; 0.4-30)	N/A

^a^N/A: not applicable.

^b^Data not available.

### Pregnant Women’s Decision-making Characteristics

All the women had made prenatal screening decisions. Most had made the final decision with a clinician (15/45, 33% with an obstetrician-gynecologist; 12/45, 27% with a midwife; 10/45, 22% with a family physician; and 1/45, 2% with both a midwife and family physician). However, some women made the decision alone (5/45, 11%) or with their partner (2/45, 4%). Most women had chosen to take the prenatal screening test (38/45, 84%), and some (28/45, 62%) of them had selected the integrated biochemical test with nuchal translucency before participating in this study.

### Quantitative Results

Table 3 shows participants’ perceptions of usability, quality, and satisfaction with the content of the computer-based DA. Mean scores of perceived usefulness and of self-efficacy for pregnant women were 80 (SD 13) and 88 (SD 10) out of 100, respectively. Table 3 also shows mean scores of usability, quality, and satisfaction with the computer-based DA for all 3 populations together (overall score) and for each one of them. The mean scores of clinicians were lower than those of pregnant women and those of policy makers. Women who were temporary or permanent residents also rated the DA lower overall than did Canadian citizens (data not shown). The mean overall usability score was 82/100 (SD 14). The mean overall quality score was 79 (SD 10) out of 100. The lowest scores were for engagement (how engaging users found the computer-based DA), especially for entertainment (mean 53, SD 22), customization (whether the computer-based DA allows the customization of settings and preferences that they would like; mean 45, SD 23), and interactivity (mean 53, SD 25). These slightly lower quality scores suggested areas for improvement in our DA (detailed in Table 3) and were the items used in our interview guide for further qualitative exploration.

The mean score for overall satisfaction with the content was 82 (SD 14) out of 100.

Table 4 shows participants’ perceptions of the acceptability of the computer-based DA. Of the 66 participants, 26 (39%) rated the presentation as “excellent,” 52 (79%) rated the amount of information as “just right,” 31 (47%) rated the worksheet as “good,” and 58 (88%) rated it “balanced.” However, 12% (8/66) of participants found that the information presented oriented users toward choosing to take the screening test. Approximately 58% (38/66) of participants found that the computer-based DA was useful, and 76% (50/66) found that the information was sufficient.

**Table 3:** Participants’ perceptions of usability, quality, and satisfaction with the content of the computer-based decision aid (N=66)^a^.

Variables				All 3 populations, mean (SD)^b^		Pregnant women (n=45), mean (SD)		Clinicians (n=13)^b^, mean (SD)		Policy makers (n=8), mean (SD)
Perceived usefulness				N/A^c^		79.9 (13.4)		N/A		N/A
Self-efficacy				N/A		88.0 (10.6)		N/A		N/A
Usability (SUS^d^)				82.6 (14.4)		83.9 (14.3)		76.5 (14.0)		79.4 (22.5)
**Quality (uMARS^e^)**										
	**Engagement**		62.7 (14.4)		64.7 (13.5)		58.4 (14.0)		57.9 (17.0)	
		Entertainment	52.9 (22.5)		55.0 (23.0)		41.7 (20.4)		60.7 (18.2)	
		Interest	82.2 (20.3)		84.4 (18.7)		71.2 (23.7)		89.3 (18.2)	
		Customizable	44.7 (23.0)		44.8 (24.2)		47.5 (18.2)		39.3 (26.2)	
		Interactivity	52.9 (24.9)		55.6 (23.8)		51.9 (18.2)		39.3 (37.5)	
		Target group	78.1 (21.4)		83.3 (18.5)		71.2 (19.2)		60.7 (29.4)	
	Functionality		90.5 (9.9)		92.4 (7.9)		82.7 (13.3)		92.9 (9.2)	
	Aesthetic		82.1 (13.6)		83.5 (12.4)		74.4 (16.5)		86.9 (11.6)	
	Information		79.6 (13.1)		81.1 (11.6)		72.4 (17.5)		83.4 (10.1)	
	Global evaluation		78.7 (9.9)		80.4 (8.9)		71.9 (11.7)		80.3 (8.7)	
Satisfaction				81.5 (13.7)		84.4 (12.6)		72.8 (16.3)		73.4 (19.8)

^a^Scale 1 to 100.

^b^Missing data=1.

^c^N/A: not applicable.

^d^SUS: System Usability Scale.

^e^uMARS: user version of the Mobile App Rating Scale.

**Table 4:** Participants’ perceptions of acceptability of the computer-based decision aid (N=66).

Dimensions of acceptability and answer choice		All 3 populations, n (%)	Pregnant women (n=45), n (%)	Clinicians (n=13^a^), n (%)	Policy makers (n=8), n (%)
**Presentation**					
	Excellent	26 (39)	20 (45)	3 (23)	3 (38)
	Good	24 (36)	15 (33)	5 (39)	4 (50)
	Fair	15 (23)	9 (20)	5 (38)	1 (12)
	Poor	1 (2)	1 (2)	0 (0)	0 (0)
**Amount of information**					
	Too little information	12 (18)	8 (18)	3 (23)	1 (12)
	Just right	52 (79)	37 (82)	9 (69)	6 (75)
	Too much information	2 (3)	0 (0)	1 (8)	1 (12)
**Worksheet**					
	Excellent	5 (8)	3 (7)	1 (8)	1 (12)
	Good	31 (47)	21 (47)	6 (46)	4 (50)
	Fair	23 (35)	17 (38)	3 (23)	3 (38)
	Poor	4 (6)	2 (4)	2 (15)	0 (0)
	N/A^b^	3 (4)	2 (4)	1 (8)	0 (0)
**Balance**					
	Slanted toward choice to be tested	8 (12)	5 (11)	2 (15.4)	1 (12)
	Slanted toward choice to not be tested	0 (0)	0 (0)	0 (0.0)	0 (0)
	Balanced	58 (88)	40 (89)	11 (84.6)	7 (88)
**Usefulness**					
	Very useful	20 (30)	14 (31)	3 (23)	3 (38)
	Useful	38 (58)	26 (58)	8 (62)	4 (50)
	Somewhat useful	8 (12)	5 (11)	2 (15)	1 (12)
	Useless	0 (0)	0 (0)	0 (0.0)	0 (0)
**Sufficient information**					
	Yes	50 (76)	38 (84)	7 (54)	5 (62)
	No	16 (24)	7 (16)	6 (46)	3 (38)

^a^Missing data=1.

^b^N/A: not applicable.

### Qualitative Results

Here, we report themes related to (1) general reaction, (2) the engagement aspects of the computer-based DA (entertainment, customization, interactivity, and target audience) as these uMARS subscales were rated lower than other subscales, (3) the questionnaire, and (4) themes emerging from responses by clinicians and policy makers (eg, strategies for implementation), along with related suggestions. We received >300 suggestions that we synthesized and grouped by theme, and we present those most relevant to usefulness and usability, along with some illustrative quotes (translated from French).

#### General Reactions

Almost all participants expressed some general reaction (64/67, 96%) to the computer-based DA. More participants gave positive comments (60/67, 90%) than negative comments (39/67, 58%). More than half (35/67, 52%) provided both positive and negative comments. The 3 most common positive comments were related to the quantity and quality of information, ease of use, and usefulness in making a decision:

It’s really well done, I was like, wow! Why didn’t we have this before? It would have helped me a lot...when I made an informed choice or even when [pregnant women] come to the information evening here.

TT-PS-SF-02

The 3 most common negative comments were that the information was too dense, incomplete, and the questionnaire was too difficult to use. Regarding density, 2 clinicians commented as follows:

It was, like, a bit overwhelming. [When I] tried to put myself in the patient’s shoes, I thought, she’d have to read it more than once to be able to fill it out...I found it heavy-going.

TT-PS-MF-03

I read it to...a friend of mine who just has a high school education but is super intelligent...and she said, “Wow that’s heavy-going, that thing, I wouldn’t even want to finish it, I’d say let’s go walk the dog instead.”

TT-PS-SF-02

Regarding incompleteness, one of the clinicians commented that chorion biopsies, not mentioned in the DA, were often performed rather than amniocenteses; and another commented that shorter wait times in the private system were missing.

#### Engagement

##### Entertainment

Of 67 participants who answered this question, 31 (46%) did not think that the computer-based DA should be too entertaining:

I don’t expect to be entertained in a jokey way when I’m looking for this kind of information. It’s not an entertaining app, but then I don’t expect to be entertained—so it’s doing a good job of providing information.

TT-GMF-33-03

Moreover, 2 (3%) participants thought it should be more entertaining:

It should be somewhat fun, so it won’t take too long. [And] then the partner might say “Can I have a go?”

TT-PS-SF-05

##### Customization

Overall, of the 67 participants, 16 (24%) thought the computer-based DA was sufficiently customizable (ie, could be adapted to users’ profiles), whereas 28 (42%) participants wanted it to be more customizable:

It’s a good idea...so people could go “okay, I want to do the nuchal scan, where’s my nearest health centre, is it in Beauce or in Quebec?” so then the couples can also decide to make an appointment, that would be great.

TT-PS-SF-06

Moreover, of the 67 participants, 21 (31%) were against any customization, and 2 (3%) were ambivalent:

It could be hurtful...if they [adapted it to my literacy level], because it’s like I’m not smart enough to understand all the information—it’s putting people in boxes, it’s a bit discriminatory.

TT-PS-SF-06

Suggestions for what could be customized included (1) geolocation (or postal code) for indicating local clinical screening sites, (2) maternal age, (3) risk factors, (4) week of pregnancy, (5) amount of information desired, or even (6) allowing customization by desired criteria (ie, *à la carte* menu). However, some were concerned about the threat of data theft:

If you open the thing and the first thing they ask is your name, your age and your postal code, I go, EW, they’re collecting data on me! I think if you don’t want to answer, you [should be able to] stay with the generic version, but if you want to personalize it, it’s your choice, and it won’t block you.

TT-DP-08

##### Interactivity

Overall, of 67 participants who answered this question, 20 (30%) thought the computer-based DA was sufficiently interactive, although 6 (9%) wanted it to be more interactive:

It’s a very linear app, there are no links to other sites, to other information...you’re in one section then you click “next” and you’re in the next section, then the next.

TT-MN-27-49

Three major suggestions for improving interactivity were (1) adding hyperlinks to other sites (eg, government sites) and relevant statistics and adding more clickable information, (2) adding a frequently asked questions section, and (3) providing a web-based chat window for live questions. The latter did not meet with unanimous approval; a few participants were against it (8/67, 12%), of whom some explained that chat agents lack credibility (3/8, 38%).

##### Target Audience

Overall, 6 clinicians or policy makers (6/67, 9%) felt that the computer-based DA would not be useful for people with high levels of anxiety and with little time, or for socioeconomically disadvantaged or uneducated clienteles. One of the clinicians explained that written information was not useful to many patients:

One in three people [have difficulty reading], even if they have a job... I’m always surprised. I give them less and less information on paper...It’s already hard to explain the risks, then risks by age...and when I call them back with the results, just to say everything’s fine, there’s easily one-third who don’t understand.

TT-PS-SF-05

Another commented that as people with limited literacy will not use it anyway, it is fine the way it is:

I think it’s simple enough for those who want to read and inform themselves on the subject, which is the vast majority, but for those who can’t read, it’ll take videos, just with “there’s this” and “there’s that.”

TT-PS-OB-02

Some thought the DA was only useful for those who were undecided and that it might be misleading:

This app is more for people who are undecided. I think when they use it, they expect that by the end it’ll make the decision for them somewhat.

TT-PS-SF-04

#### Values and Preferences Questionnaire

Most participants thought that the statements (benefits and risks of undergoing a screening test or not) were difficult to understand (40/67, 60%) and that the 1 to 10 scale was difficult to use (13/67, 19%):

I found it a bit vague, it was too much...I’m okay with proportions, but I’m not that comfortable—so I had to read the question two or three times...So I’m not likely to say to someone else “Do this questionnaire, it’s really helpful.” Because even when I’d finished a question, I still wasn’t sure if I’d answered it properly.

TT-GMF-29-38

Although the DA only summarizes users’ answers, half of the pregnant women (23/45, 51%) expected it to direct them to a choice based on the information they had provided. At the same time, a large proportion (27/45, 60%) did not want the DA to guide them to a specific choice:

I think it’s good that it doesn’t tell you yes or no you should do it...But imagining myself as a woman who’s not sure—then at the end it just tells you what you’ve already said...Then you’re, like, so should I do it or not? I think it’s good...that it doesn’t guide people too much.

TT-GMF-30-27

Suggestions by women, clinicians, and policy makers for improving the questionnaire were to (1) use decision trees, (2) use a visual diagram to summarize the weight of each advantage and disadvantage, (3) present the questionnaire results in the form of a “compass” that analyzes user responses to help them position themselves among the options [34], (4) give users a simpler way of weighing the benefits against the risks, (5) show the general direction of the person’s choices, (6) show users’ prioritized advantages and disadvantages in the order of importance in the summary table, (7) highlight gray areas (score of 4, 5, and 6) to indicate that users should discuss it with their health care provider, (8) color-code the factors assigned an importance of 6 to 10, and (9) use a simpler rating system than a 10-point scale.

#### Additional Themes Raised by Clinicians and Policy Makers

##### Perceptions of Usefulness

Overall, 6 clinicians or policy makers thought that the computer-based DA would be used by >50% of their colleagues but that not everyone would be comfortable using an app (ie, added support would be needed for vulnerable women):

For sure, people...who have fairly limited literacy, the concepts with initials [abbreviations], and the prevalence rates, all that this person would need to be accompanied by...a health professional to understand what the impacts are, the advantages, and disadvantages.

TT-DP-17

However, the health professional who would accompany the woman would also need to be fully informed:

The shortcomings we come back to are about when the person returns to the professional. Yes the professional has the expertise, has probably been trained to welcome the pregnant woman and discuss [testing] with her correctly, present the options. But from what we have seen, there is so much to remember...to be sure they re-train now and then, when new techniques and/or consent practices have evolved.

TT-DP-15

When clinicians were asked whether the information was what their patients needed, most (5/14, 36%) said that it was similar to the information they offered and that they sometimes gave more, such as on markers, on available tests privately, on chorionic biopsy, and on the fact that screening detects trisomy but other anomalies cannot be detected yet.

##### Implementation of the Computer-Based DA in Clinical Practice

Policy makers all thought that clinicians should integrate SDM into their practice and recommended that the use of computer-based DA should be incorporated into practice guidelines and into continuing professional education for clinicians.

They also recommended providing a link or number for women to call if they had questions and suggested conducting a DA implementation pilot project followed by rollout on a large scale.

Clinicians (11/14, 79%) thought that the computer-based DA should be promoted and given to pregnant women and their partners as early in pregnancy as possible: (1) before their first consultation with the prenatal care specialist, (2) during the information session at approximately 8 to 10 weeks at the birthing center, (3) at approximately 5 to 8 weeks during the meeting with the nurse, (4) at between 7 and 10 weeks to give parents time to prepare for the decision, (5) by phone during the first call with the secretaries who would refer them to a download link, or (6) when the physician sends a prescription for nuchal translucency (if there is no consultation before testing). They also thought that the computer-based DA should not be used during consultations but during a later encounter to discuss any questions it may have raised (6/14, 43%):

[A nurse] could tell them to go on the site, go through the process, and then talk about it...it could be repeated with the doctor to assimilate the information, they could talk to their partner, prepare specific questions, etc...Most of our doctors see them around 10 weeks, that’s when we have to prescribe what to do—like, are we doing [the test], or not? But if the process is all done in the doctor’s office, it will never end. Doctors won’t get on board with that, I don’t think.

TT-PS-MF-03

#### Other Suggestions

Participants also suggested (1) using more neutral and unisex colors; (2) using a denominator smaller than 10,000 for the presentation of risk by age; (3) using video clips instead of text; and (4) collaborating with pregnancy tracking applications, which could include a link to the computer-based DA and send a notification to users when it is time to make a decision.

## Discussion

### Principal Findings

We assessed the usability and usefulness of a computer-based DA among pregnant women, clinicians, and policy makers. Participants found that it improved self-efficacy for decision-making, was helpful for preparing for decision-making, was usable, and was of good quality overall. They were also satisfied with its content, and based on the scores for the various dimensions of acceptability, the computer-based DA was also found to be acceptable. In the qualitative interviews, the participants were mostly positive but less so about how engaging the app was. They made suggestions for improving the questionnaire and proposed implementation strategies.

First, participants reserved their lowest scores for engagement. They proposed that more advanced digitization features, such as customization and interactivity, would make it more engaging. Customization is necessary for better culturally adapted DAs [35] and to avoid information overload [36]. In another screening context (colorectal cancer), a computer-based DA was customizable for age and gender, and participants were asked for further customizable features such as family history and medical history [37]. However, some types of customizability are easier to operationalize (eg, age) than others (eg, geographic location) as the latter requires continuous updating of the registry of clinical sites available for screening. This would require input from the Ministry of Health and Social Services, which holds this registry [38]. In addition, a geolocation feature, whereby users would provide personal data such as their postal or zip code, poses a privacy risk [39].

Second, the study participants had difficulty in both using the values clarification questionnaire and interpreting the results. In a previous study evaluating an earlier, paper-based version of this DA [22], participants also had difficulty using a values clarification exercise with 5 rating stars, with 1 meaning “not important” and 5 meaning “very important” [40]. This suggests that we explore values clarification methods that simply offer users options without asking them to measure their importance to them on a scale (ie, users choose the elements they wish to consider before deciding whether to do the test). Participants also highlighted the difficulties they encountered in interpreting the results after completing the values clarification exercise. Moreover, most pregnant women expected the DA to make the choice for them based on the information they provided. When faced with a difficult decision, the human tendency is to offload it onto someone (or something) else, especially when the choices have potentially negative consequences [41]; however, the use of mHealth should not remove users’ responsibility for the decision. For a DA or clinician to make the decision for them would go against the principle of empowerment conveyed through their active participation in SDM [42,43]. If the expectation of a ready-made decision was raised by the computer-based DA itself, it will be stated more clearly on the home page that the DA will provide them only with the elements to make their decision.

Finally, women who were Canadian citizens were more satisfied with the content of the computer-based DA than temporary or permanent residents. It is very likely that this explanation lies in the diverse cultures of immigrants and their language limitations. Further research is needed to understand immigrant women or couples’ expectations of and attitudes toward the DA. This difference in satisfaction demonstrates the importance of developing a culturally sensitive DA, such as translating it into other languages.

### Limitations

This study had several limitations. First, we recruited women after they had already made their screening decisions. They had to imagine that they were still in the situation of making the decision to answer the questions. This may have biased our results. However, the time between their decisions and the study was relatively short. Second, in Canada, prenatal care requires the collaboration and coordination of many different health care providers, including nurses, who were not involved in the study [44]. However, approximately 98% of pregnancies are monitored by the types of clinicians involved in this study [45,46]. Third, education level and household income were higher in our sample than in the general population. However, the participants mentioned that our DA needed to be adjusted for use by less-educated women. Finally, we did not meet our sample size requirements for clinicians and policy makers. However, their experience provided important data on how to improve and implement the computer-based DA in primary care settings.

### Conclusions

We assessed the usability and usefulness of a computer-based DA among pregnant women, clinicians, and policy makers. They informed us that the tool could be improved with more customization options, more interactivity, and a simpler value clarification exercise. The next step will be to incorporate participants’ suggestions and implement the computer-based DA in primary care settings across Quebec prenatal care clinics.

## Appendix

Multimedia Appendix 1 Standards for Universal reporting of patient Decision Aid Evaluation studies (SUNDAE) checklist.

## Abbreviations

DA: decision aid
mHealth: mobile health
MMARS: Mixed Methods Article Reporting Standards
SDM: shared decision-making
SUNDAE: Standards for Universal reporting of patient Decision Aid Evaluation studies
uMARS: user version of the Mobile App Rating Scale

## Data Availability

The data sets used and analyzed during the study are available from the corresponding author upon reasonable request.
